# CD8 T cell function and cross-reactivity explored by stepwise increased peptide-HLA versus TCR affinity

**DOI:** 10.3389/fimmu.2022.973986

**Published:** 2022-08-10

**Authors:** Petra Baumgaertner, Julien Schmidt, Carla-Marisa Costa-Nunes, Natacha Bordry, Philippe Guillaume, Immanuel Luescher, Daniel E. Speiser, Nathalie Rufer, Michael Hebeisen

**Affiliations:** ^1^ Department of Oncology, Lausanne University Hospital and University of Lausanne, Epalinges, Switzerland; ^2^ Ludwig Institute for Cancer Research, Lausanne Branch - University of Lausanne, Epalinges, Switzerland

**Keywords:** cancer immunotherapy, vaccine peptide, melan-A/MART-1, NY-ESO-1, peptide-HLA binding affinity, TCR-peptide-MHC affinity, human CD8 T cells

## Abstract

Recruitment and activation of CD8 T cells occur through specific triggering of T cell receptor (TCR) by peptide-bound human leucocyte antigen (HLA) ligands. Within the generated trimeric TCR-peptide:HLA complex, the molecular binding affinities between peptide and HLA, and between TCR and peptide:HLA both impact T cell functional outcomes. However, how their individual and combined effects modulate immunogenicity and overall T cell responsiveness has not been investigated systematically. Here, we established two panels of human tumor peptide variants differing in their affinity to HLA. For precise characterization, we developed the “blue peptide assay”, an upgraded cell-based approach to measure the peptide:HLA affinity. These peptide variants were then used to investigate the cross-reactivity of tumor antigen-specific CD8 T cell clonotypes derived from blood of cancer patients after vaccination with either the native or an affinity-optimized Melan-A/MART-1 epitope, or isolated from tumor infiltrated lymph nodes (TILNs). Vaccines containing the native tumor epitope generated T cells with better functionality, and superior cross-reactivity against potential low affinity escape epitopes, as compared to T cells induced by vaccines containing an HLA affinity-optimized epitope. Comparatively, Melan-A/MART-1-specific TILN cells displayed functional and cross-reactive profiles that were heterogeneous and clonotype-dependent. Finally, we took advantage of a collection of T cells expressing affinity-optimized NY-ESO-1-specific TCRs to interrogate the individual and combined impact of peptide:HLA and TCR-pHLA affinities on overall CD8 T cell responses. We found profound and distinct effects of both biophysical parameters, with additive contributions and absence of hierarchical dominance. Altogether, the biological impact of peptide:HLA and TCR-pHLA affinities on T cell responses was carefully dissected in two antigenic systems, frequently targeted in human cancer immunotherapy. Our technology and stepwise comparison open new insights into the rational design and selection of vaccine-associated tumor-specific epitopes and highlight the functional and cross-reactivity profiles that endow T cells with best tumor control capacity.

## Introduction

Cytotoxic CD8 T cells play key roles in generating protective and durable immune responses against pathogens and cancer. The specificity of CD8 T cell responses relies on the recognition by T cell receptors (TCR) of small immunogenic peptides restricted by major histocompatibility complex (MHC) molecules - termed Human Leukocyte Antigens (HLA) in humans - at the surface of antigen presenting cells (APC). Multiple factors and regulatory mechanisms control the presence and function of CD8 T cells within tumors, greatly influencing disease outcome in numerous human malignancies (1). These factors can be T cell-extrinsic (e. g. inflammation and suppressive mechanisms in the tumor microenvironment, amount of tumor-derived neoantigens) (2, 3) or T cell-intrinsic (e. g. reactivity of T cells to tumor antigens/neoantigens, stem-like vs exhausted differentiation states, functional avidity and proliferation capacities), all impacting on how tumor-specific T cells will recognize and control/eliminate tumor cells (4, 5). Molecularly, many T cell-intrinsic factors initially depend on the interaction strength occurring between the tumor-specific TCR and the peptide:HLA antigen (i.e. the so-called TCR-pHLA affinity) (6, 7), which is itself conditioned by the interaction characteristics of the antigenic peptide within the HLA binding groove (i.e. the peptide:HLA affinity) (8, 9). As such, both biophysical parameters within the TCR-peptide:HLA complex are key determinants of TCR triggering sensitivity and subsequent T cell responses, referred to as “ T cell functional avidity” (i.e. EC_50_: the concentration of peptide producing half-maximal T cell responses during *in vitro* functional titration assay). Since functional avidity represents a major hallmark of T cell-associated tumor clearance (10), its systematic assessment in detailed cytokine or killing assays represents a crucial readout for categorizing the tumor cell recognition capacity of distinct cytotoxic T cells.

Natural tumor-reactive T cells mostly express TCRs of low affinity for self/tumor epitopes (11). This is because central and peripheral tolerance eliminate and control potentially toxic cross-reactive T cells of high affinity against self-antigens. In contrast, immune responses to non-self antigens (e.g. viral) are dominated by cytotoxic T cells expressing high affinity TCRs, which are functionally superior than low affinity T cells (12, 13). T cells recognizing tumor-derived neo-epitopes have functional avidities between the ranges of self- and non-self, depending on the structural similarity or dissimilarity of the neo-epitope to self-antigens (14). To improve efficacy of tumor recognition in adoptive T cell immunotherapy, T cells were engineered to express affinity-enhanced TCRs against various tumor-associated antigens (15). Functional analysis highlighted that optimization of T cell responses against cancer was achievable when augmenting TCR affinity toward values observed for non-self TCR, but at the cost of enhanced auto- and cross-reactivity (16–19). Furthermore, engineering T cells with TCRs above the upper natural affinity threshold triggered chronic TCR-HLA-A2 self-reactivity even in the complete absence of cognate antigen (20). This generated T cells with an initial hyperactive state (20), followed by a long-term tolerance/exhausted-like hyporesponsiveness due to negative feedback mechanisms, resulting in severe functional decline (21, 22). On the other end of the spectrum, T cells with very low affinity TCRs, although potentially reacting rapidly to tumor antigens, are not useful because they fail controlling disease and preventing tumor relapse (23). New strategies are currently being explored to develop high killing potency T cells with minimal TCR affinity-associated toxicity (24).

The characteristics and binding strength of the antigenic peptide within the HLA binding groove (i.e. peptide:HLA affinity) also impacts on the biological outcome and functional avidity of T cells (10, 25, 26). Altered peptide variants (also referred to as mimotopes or heteroclitic peptides) with increased affinity for MHC/HLA showed superior T cell activation potential and tumor cell control than the native peptide antigen in mouse models (27–29), prompting their use in clinical trials (30). However, vaccination with high affinity peptide variants, even if recruiting higher fractions of T cells with strong cytotoxic profiles, often remained therapeutically inefficient in human. This is because reactivity of such T cells toward the native, endogenous tumor antigen became limited, suggesting vaccination-dependent biases in TCR repertoire associated to altered cross-reactivity potential (13, 31). To assess the affinity of ligands to HLA, two types of experimental designs have been developed: 1) cell-free peptide:HLA stability/dissociation assays and 2) cell-based binding/functional competitive assays, which would be superior in predicting real peptide immunogenicity (32, 33). In the last decade, with the increasing number of peptide:HLA structural complexes and mass-spectrometry-based targets deposited in open-source platforms, computational-based algorithms were developed to calculate peptide:HLA stability and affinity with the ultimate goal of predicting the immunogenicity of the entire HLA peptidome (34). Although highly attractive for the field of tumor immunology and vaccination, finding tumor-specific antigens with high HLA binding score based on prediction algorithms does not currently grant immunogenicity toward endogenous targets and potential escape derivatives. This also depends on additional parameters, such as expression and presentation of the antigen, turnover, accessibility and affinity of the complex to TCR (35). Indeed, strong divergences between prediction and assay-based functional and immunogenicity analyses were detected for series of endogenous cancer neo-epitopes (36–38), as well as for NY-ESO-1-derived 8 to 11-mers bound to HLA-A2*01 (39). Therefore, novel T cell epitope prediction algorithms are currently being developed to integrate additional biophysical parameters (40, 41).

In this study, we developed a peptide:HLA binding affinity assay (defined as the “blue peptide assay”) to assess a panel of peptide variants with a large spectrum of class I HLA binding capacities in two tumor antigen-specific CD8 T cell model systems relevant for cancer vaccination and adoptive cell transfer immunotherapy. We first explored the cross-reactivity profile of human CD8 T cells recruited after vaccination with either the native or an affinity-optimized Melan-A/MART-1_26-35_ peptide and found better control of potential escape epitopes by T cells isolated from patients vaccinated with the native tumor antigen. We then compared those results to the heterogenous cross-reactive profiles obtained from tumor-primed T cell clones derived directly from TILNs. Finally, we asked whether the functional avidity was dominantly controlled by the peptide:HLA binding strength or the TCR-pHLA affinity and found that neither was the case. By testing and analyzing stepwise combinations of increased peptide:HLA affinity (K_i_) and TCR-pHLA affinity (K_D_), we here dissected the molecular interactions within the trimolecular TCR-pHLA complex that influence T cell responses and cross-reactivity against potential escape epitopes.

## Material and methods

### Ethics approval

Study protocol (LUD00-018) was designed, approved, and conducted according to the relevant regulatory standards from (i) the Ethics Committee for Clinical Research of the University of Lausanne (Lausanne, Switzerland), (ii) the Protocol Review Committee of the Ludwig Institute for Cancer Research (New York), and (iii) Swissmedic (Bern, Switzerland). Patient recruitment, study procedures, and blood withdrawal were carried out upon written informed consent prior to study inclusion. Human peripheral blood cells were obtained from healthy donors of the Interregional Blood Transfusion SRC Ltd. All blood donors had previously completed the Swiss National Medical questionnaire to verify that they fulfilled the criteria for blood donation and provided written informed consent for the use of blood samples in medical research after anonymization.

### Culture of cell lines and primary CD8 T lymphocytes

Melan-A specific CD8 T cell clones were generated from melanoma patients included in the phase I clinical study LUD00-018 (ClinicalTrials.gov Identifier NCT00112229). Antigen specific CD8 T cells were sorted *ex vivo* from patient PBMC or TILNs with phycoerythrin-labeled HLA-A*02:01/Melan-A/MART-1 A27L peptide_26-35_ tetramer (peptide & tetramer core facility, Department of Oncology, UNIL) and CD8 APC-Cy7 (BD Bioscience). Individual clones were obtained by limiting dilution (0.5 cell/well) in Terasaki plates and cultured in RPMI medium with 8% HS and 150 U/ml IL-2, 10000 irradiated allogenic feeder cells per well and 1 μg/ml PHA. The clones were tested for antigen specificity by tetramer staining. TAP (transporter associated with antigen processing)-deficient T/B hybrid HLA-A2^pos^ T2 cells were used as antigen presenter cells. T2 cells are defective in processing endogenous self-peptides, but able to present exogenously-pulsed peptides efficiently (42). C1R (deficient in HLA-A and B expression (43), C1R-A2 and -A3 transduced cells were used for assessing correct HLA restriction of the blue peptide. C1R and TAP-deficient T2 cells were cultivated in RPMI%10% FCS, Penicillin and Streptomycin and maintained at 37°C and 5% CO_2_.

### NY-ESO-1–specific TCR αβ constructs, lentiviral production, and cell transduction

Cloning strategies and lentiviral production were performed as described previously (44, 45). The full-length codon-optimized TCR AV23.1 and TCR BV13.1 chain sequences of a dominant NY-ESO-1_157–165_–specific T cell clone of patient LAU155 were cloned in the pRRL, third generation lentiviral vectors, as an hPGK-AV23.1-IRES-BV13.1 construct. Structure-based amino-acid substitutions were introduced into the WT TCR sequence using the QuikChange Mutagenesis Kit (Stratagene) and confirmed by DNA sequencing. Supernatant of lentiviral-transfected 293T cells was used to infect primary CD8 T lymphocytes. PE-labeled A2/NY-ESO-1_157–165_–specific multimers were used to sort transduced primary CD8 T cells to purity. Integrated lentiviral copy number was found to be 1-2 copies/genome for each TCR variants.

### Synthesis of unlabeled and blue peptides

Peptides were synthesized by the Peptide and Tetramer Core Facility, UNIL-CHUV, Epalinges, Switzerland, by standard solid phase peptide synthesis on a multiple peptide synthesizer (Intavis, Germany). All peptides were > 90% pure as indicated by UPLC-MS analysis. Lyophilized peptides were diluted in pure DMSO at 10 mg/ml and stored at -80°C (stock solution). 10x diluted aliquots (1 mg/ml in 10% DMSO) were prepared from the stock and used for killing assay. Cy5-labeled HBVc peptide (blue peptide) was prepared by alkylation of the cysteine in position 6 of the native peptide with maleimide-Cy5 (Pierce, Thermo Fisher Scientific) in Tris 0,1 M pH 7, for 2 h. The labeled peptide was purified by RP-HPLC, analyzed by UPLC-MS and kept lyophilized at -80°C.

### *In silico* prediction of peptide binding strength to MHC class I

The potential binding strength of the NY-ESO-1_157-165_ and Melan-A_26-35_ peptide variants was determined *in silico* with IEDB analysis resource Consensus tool (http://tools.iedb.org/mhci/) (46), NetMHC 4.0 prediction algorithm (https://services.healthtech.dtu.dk/service.php?NetMHC-4.0) (34) and the latest PRIME2.0 algorithm (http://prime.gfellerlab.org) (41).

### Peptide-driven soluble refolding assay

A peptide-driven soluble refolding assay was used to assess the molecular stability of peptide:HLA complexes in a cell-free environment. Refolding with HLA-A0201 heavy chain carrying a C-terminal BirA substrate peptide (BSP), biotin-labeled β2m and a test peptide were performed essentially as described (47). Human β2m was mutated at S88C and after refolding, alkylated with maleimide-PEG_2_-biotin (Pierce, Thermo Fisher Scientific) in PBS at pH 7.4. Refolding reactions were performed in 96 well plates at 4°C for 72 h in the presence of 10 µM peptide. Incubation without peptide and with the Flu matrix_58-66_ peptide served as negative and positive controls, respectively. After centrifugation (4’000 rpm, 5 min), the reaction mixtures were transferred into 96 well plates coated with anti-BSP antibody and saturated with 1% BSA. After 2 h, plates were washed 5 times with PBS-Tween 0,05% and extravidin-alcaline phosphatase (Sigma) was added. After 1 h at room temperature, plates were washed 5 times with PBS-Tween 0,05% and PNPT substrate was added (Sigma). OD at 405 nm was read after 20 min on a 96 well plate reader using the Gen5 software. All measurements were performed in triplicates and normalized to Flu matrix_58-66_ value, which was set at OD = 1.

### Blue peptide (Cy5-labeled HBVc) kinetic and isotherm determination

The binding equilibrium dissociation constant (K_D_) of the Cy5-labeled HBVc blue peptide on HLA-A2 molecules was found through parallel binding association (k_on_) and dissociation (k_off_) assessment, as well as binding equilibrium assays using HLA-A2^pos^ and HLA-A2^neg^ presenting cells at 4°C and at 37°C. In short, for k_on_ measurements on T2 (HLA-A2^pos^) and C1R-A3 (HLA-A2^neg^) cells, baseline Cy5 cell auto-fluorescence was recorded for 30s with an LSRII instrument. Titrated amounts of Cy5-labeled HBVc blue peptide (from 1 pM to 1 uM final concentration) were then added to the tube and association of the blue peptide to HLA-A2 was recorded for 5 minutes. For k_off_ dissociation assays, 2x10^6^ HLA-A2^pos^ (T2) and HLA-A2^neg^ (C1R-A3) cells were stained with 1 μM blue peptide and incubated for 1h in the dark. After washing, baseline Cy5 staining was recorded for 30s, before adding excess of FACS buffer and recording fluorescence intensity (gMFI) for 1h under temperature control. Additionally, isotherm titration experiments were performed to validate the value of the blue peptide dissociation equilibrium constant K_D_. Cells were stained with titrated amounts of the blue peptide (from 1 pM to 1 uM) for 1h at 4°C or 37°C. After washing, Cy5 gMFI was recorded on an LSRII instrument to determine concentration-associated maximal staining at equilibrium conditions (n = 5). All kinetic and isotherm analyses were done with the corresponding best fit equations using Prism software (GraphPad, v.9.1.1) and correction of non-specific staining values found on control HLA-A2^neg^ cells (nonlinear regression for k_on_ = association kinetics - one conc. of ligand, for k_off_ = dissociation/one phase exponential decay and for K_D_ = one site - total and nonspecific binding).

### Competition assay and K_i_ calculation

TAP-deficient HLA-A2^+^ T2 cells were incubated for 1h at 37°C and 5% CO_2_ with anti-HLA-class I antibody W6/32 in RPMI at a concentration of 1 μg/ml to stabilize the MHC-complex. The cells were washed and resuspended at 0.5x10^6^ cells/ml for the test in RPMI/10% FCS and β2-microglobilin at a final concentration of 1.5μl/ml. Meanwhile, a serial dilution from 50μM to 0.1μM of the “competitor peptide” (peptide of interest) was prepared in pure RPMI. The Cy5-labeled HBVc blue reference peptide was prepared at a fixed concentration of 0.2 μM final in pure RPMI to generate a sub-saturating MFI signal of 80% of the maximum, which was found to provide optimal sensitivity for peptide competition. Both, the competitor and the reference peptides were incubated with the T2 cells for 4h at 37°C and 5% CO_2_. Cy5 fluorescence of the blue-HBVc peptide bound on T2 after competition with titrated dose of the competitor peptide was acquired by Flow Cytometry (BD FacsArray). The data were analysed with the FlowJo 9.9.4 software. IC50 and K_i_ values were interpolated using the one site Fit logIC50 or the one site Fit K_i_ nonlinear regression equations using Prism software (GraphPad, v.9.1.1) under Cheng & Prusoff conditions (48) with constant blue peptide concentration = 200 nM and K_D_ = 52.6 nM.

### Killing assay

The specific lytic activity of the NY-ESO-1 CD8 T-cell lines or Melan-A specific T cell clones was assessed by presentation of HLA-A*02 peptide variants of NY-ESO-1_157-165_ and Melan-A_26-35_ on ^51^Cr-labeled TAP-deficient T2 cells. T2 cells were labeled with ^51^Chromium (Amersham Biosciences) for 1h, washed and resuspended in culture medium. ^51^Cr-labeled cells were then incubated for 4h at 37°C with effector T cells at an Effector : Target ration of 10:1 with titrated amount of NY-ESO-1_157-165_ and Melan-A_26-35_ peptide variants At the end of the incubation time, the supernatants were harvested and radioactivity was counted in an automatic gamma-counter TopCount NXT (Perkin-Elmer). The percentage of specific lysis was determined using the formula: (experimental-spontaneous release)/(maximum-spontaneous) x 100. Internal controls were included in each assay to measure the spontaneous release (target cells alone) and the total release (target cells with 1 M HCl) (49).. Functional avidity (EC_50_ = peptide concentration giving 50% maximal killing) was derived from the nonlinear log(agonist) vs. response equation using Prism software (GraphPad, v.9.1.1).

### Statistical analysis

Correlations and statistics were performed using Prism software (GraphPad, v.9.1.1). Correlations are given as R squared values from original Pearson analysis. Regression lines were derived from nonlinear least square fitting values using straight or LOG-LOG lines depending on the XY axes. Slopes (ΔY/ΔX) correspond to the steepness of the regression lines. Statistical analyses between native (EAA) and analog (ELA) EC_50_ values and between linear regression slopes were obtained following Mann-Whitney nonparametric tests. Two-tailed *p* values were defined with a 95% confidence level. Significance of the adjusted *p* value at α = 0.05 is given by the following symbols: ns (*p* > 0.05), *p ≤ 0.05, **p ≤ 0.01, ***p ≤ 0.001, ****p ≤ 0.0001.

## Results

### Selection of a panel of Melan-A/MART-1 and NY-ESO-1-specific peptide variants displaying variable HLA binding affinities

**Figure 1:**
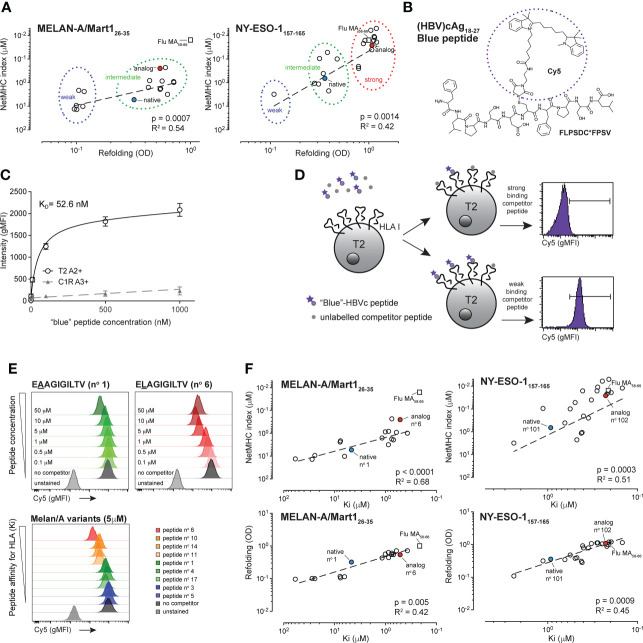
Biophysical characterization of the HBV-specific blue peptide and selection of Melan-A/MART1_26-35_ and NY-ESO-1_157-165_ -specific peptide variants.**(A)** Correlation between NetMHC4.0 and binding assay refolding indexes: Melan-A_26-35_ peptide variants (left graph) segregate into weak (blue circle) and intermediate (green circle) binding peptides. NY-ESO-1_157-165_ peptide variants (right graph) segregate into weak (blue circle), intermediate (green circle) and strong (red circle) binding peptides. Native and analog peptides are shown in blue and red, respectively. Flu Matrix_58-66_ control peptide is shown for comparison purposes (square). R square and p values were obtained from Pearson correlation analysis. **(B)** Chemical structure and sequence of the HLA-A2-restricted Hepatitis B virus (HBV) cAg_18-27_ (FLPSDC*-(Cy5) FPSV) blue peptide. **(C)** Representative experiment from concentration isotherm assays to characterize the K_D_ binding affinity of the HBVc-Cy5 blue peptide for HLA-A2 (n = 5). **(D)** Principles of the HBVc-Cy5 blue peptide cell-based competition assay. The binding strength of peptide variants to HLA-A2 complex was determined through competition assays on T2 cells between titrated amount of unlabelled competitor peptide and a fixed concentration of HBVc-Cy5 blue peptide. Examples from competition with a strong or a weak binding peptide are shown. **(E)** Blue peptide assay readouts showing titration Cy5 gMFI histograms (upper panels) at different Melan-A peptide concentrations (native EAAGIGILTV (n°1) and analog ELAGIGILTV (n°6) are shown) and with competition peptide variants having distinct affinities for HLA-A2 (lower panel). Positive control is done with the “blue”-HBVc-Cy5 peptide alone and negative control corresponds to the autofluorescence of T2 cells. **(F)** Correlation between K_i_ affinities (μM) obtained from the cellular blue peptide competition assay and values obtained from the soluble refolding assay (OD) or from the NetMHC4.0 algorithm for both NY-ESO-1 (left) and Melan-A/MART1 (right) variants. Native and analog peptides are shown in blue and red for both peptides, respectively. Flu Matrix_58-66_ control peptide is shown for comparison purposes (square).

To study the impact of the binding strength of peptide to HLA molecules on the functional responses of human tumor-reactive CD8 T cells, we generated two panels of HLA-A*0201-restricted peptide variants of the tumor-associated (TA) antigen Melan-A/MART-1_26-35_ (EAAGIGILTV) and the cancer testis (CT) antigen NY–ESO-1_157-165_ (SLLMWITQC) native peptides (50, 51). Peptide binding to HLA-A*0201 is known to depend mainly on the identity of the amino-acid at the dominant anchor positions P2 and P9/10 (52). Translating knowledge from functional alanine and substitutions scans (**Supplementary Figures 1A, B**) (44, 53, 54), we modified these two positions to generate multiple combinations of P2 and P9/10 substitution variants for both Melan-A/MART-1_26-35_ (EAAGIGILTV) and NY–ESO-1_157-165_ (SLLMWITQC) peptides. Our aim was to generate peptide variants able to bind HLA-A*0201 with altered affinities and to trigger T cell activation. We selected a total of 17 Melan-A/MART-1_26-35_ and 21 NY–ESO-1_157-165_ variants covering maximal peptide:HLA binding range (**Table 1** and **2**), which clustered into weak, intermediate and strong HLA binders, as determined by soluble refolding measurements (**Figure 1A**). Notably, Melan-A/MART-1-specific variants showed mostly weaker molecular stabilities than NY–ESO-1_157-165_ variants. For both antigenic variant panels, the refolding values correlated with the algorithm-based NetMHC4.0, IEDB and PRIME2.0 peptide:MHC binding indexes (**Figure 1A** and **Supplementary Figure 2A**), values expected to be associated with epitope presentation quality and immunogenicity (34, 41).

**Table 1:** Sequences, algorithm-based and biophysical values of HLA-A*0201-restricted Melan-A/MART1_26-35_ P2/P10 peptide variants and Flu Matrix_58-66_ control.

Melan-A/MART-1_26-35_							
peptide variant	Sequence (decamer)	IEDB pred.	NetMHC 4.0	PRIME 2.0	refolding assay	Peptide IC50	Ki affinity*
n°	P2 P10	% rank	μM	% rank	O.D.	μM	μM
1 (native)	E-**A**-A-G-I-G-I-L-T-**V**	13.5	5.32	0.74	0.32	22.50	4.67
2	E-**A**-A-G-I-G-I-L-T-**L**	22	9.30	0.92	0.10	117.00	24.47
3	E-**A**-A-G-I-G-I-L-T-**A**	24	13.78	1.68	0.10	133.00	27.68
4	E-**A**-A-G-I-G-I-L-T-**I**	30	10.21	1.44	0.11	30.70	6.39
5	E-**A**-A-G-I-G-I-L-T-**M**	30	13.14	2.25	0.10	284.00	59.14

6 (analog)	E-**L**-A-G-I-G-I-L-T-**V**	1.8	0.25	0.11	0.54	2.48	0.52
7	E-**M**-A-G-I-G-I-L-T-**V**	4.1	0.23	0.26	0.60	3.60	0.75
8	E-**I**-A-G-I-G-I-L-T-**V**	6.65	1.93	0.28	0.54	3.35	0.70
9	E-**V**-A-G-I-G-I-L-T-**V**	6.7	3.15	0.36	0.63	4.21	0.88

10	E-**L**-A-G-I-G-I-L-T-**L**	1.9	0.82	0.14	0.44	4.17	0.87
11	E-**L**-A-G-I-G-I-L-T-**A**	4.95	1.60	0.32	0.43	5.61	1.17
12	E-**L**-A-G-I-G-I-L-T-**I**	4.5	0.91	0.26	0.71	2.93	0.61
13	E-**L**-A-G-I-G-I-L-T-**M**	7.15	2.14	0.47	0.10	36.80	7.66

14	E-**M**-A-G-I-G-I-L-T-**L**	4.45	0.86	0.34	0.56	4.38	1.01
15	E-**M**-A-G-I-G-I-L-T-**A**	7.3	1.79	0.64	0.55	3.62	0.75
16	E-**M**-A-G-I-G-I-L-T-**I**	7.85	0.98	0.54	0.71	1.79	0.37
17	E-**M**-A-G-I-G-I-L-T-**M**	8.9	2.40	0.94	0.12	37.50	7.81

FluMA_58-66_	G-I-L-G-F-V-F-T-L	0.80	0.016	0.006	1.00	1.02	0.21

* Blue peptide KD = 52.6 nM.

**Table 2:** Sequences, algorithm-based and biophysical values of HLA-A*0201-restricted NY-ESO-1_157-165_ P2/P9 peptide variants and Flu Matrix_58-66_ control.

NY–ESO-1_157-165_							
peptide variant	Sequence (nonamer)	IEDB pred.	NetMHC 4.0	PRIME 2.0	refolding assay	Peptide IC50	Ki affinity*
n°	P2 P9	% Rank	μM	% Rank	O.D.	μM	μM
101 (native)	S-**L**-L-M-W-I-T-Q-**C**	3.4	0.66	0.41	0.36	4.45	0.93
102 (analog)	S-**L**-L-M-W-I-T-Q-**A**	0.7	0.03	0.18	1.08	1.09	0.23
103	S-**L**-L-M-W-I-T-Q-**L**	0.4	0.02	0.04	0.91	1.97	0.41
104	S-**L**-L-M-W-I-T-Q-**V**	0.2	0.01	0.04	1.05	1.19	0.25
105	S-**L**-L-M-W-I-T-Q-**M**	1.3	0.05	0.28	0.38	2.38	0.50
106	S-**L**-L-M-W-I-T-Q-**I**	0.5	0.02	0.10	1.13	1.36	0.28

107	S-**A**-L-M-W-I-T-Q-**A**	7.3	2.25	1.19	0.39	2.28	0.47
108	S-**A**-L-M-W-I-T-Q-**L**	4.1	1.07	0.43	0.28	1.99	0.41
109	S-**A**-L-M-W-I-T-Q-**V**	2.9	0.25	0.36	0.79	1.70	0.35
110	S-**A**-L-M-W-I-T-Q-**M**	10	3.12	1.69	0.11	11.40	2.38
111	S-**A**-L-M-W-I-T-Q-**I**	5.8	1.15	0.73	0.31	2.03	0.42

112	S-**M**-L-M-W-I-T-Q-**A**	0.7	0.02	0.38	1.20	0.95	0.20
113	S-**M**-L-M-W-I-T-Q-**L**	0.4	0.01	0.11	1.13	1.34	0.28
114	S-**M**-L-M-W-I-T-Q-**V**	0.2	0.01	0.09	1.19	1.01	0.21
115	S-**M**-L-M-W-I-T-Q-**M**	1.8	0.04	0.56	1.07	1.57	0.33
116	S-**M**-L-M-W-I-T-Q-**I**	0.7	0.01	0.21	1.15	0.71	0.15

117	S-**I**-L-M-W-I-T-Q-**A**	2.7	0.21	0.50	0.78	1.92	0.40
118	S-**I**-L-M-W-I-T-Q-**L**	1.3	0.10	0.15	0.32	5.42	1.13
119	S-**I**-L-M-W-I-T-Q-**V**	0.5	0.02	0.13	0.88	1.06	0.22
120	S-**I**-L-M-W-I-T-Q-**M**	4.9	0.41	0.73	0.35	3.10	0.64
121	S-**I**-L-M-W-I-T-Q-**I**	2.2	0.10	0.29	0.48	2.70	0.56
							
FluMA_58-66_	G-I-L-G-F-V-F-T-L	0.80	0.016	0.006	1.00	1.02	0.21

* Blue peptide KD = 52.6 nM.

### The blue peptide assay, an upgraded cellular competition assay to assess peptide:HLA binding strength on living cells

Although useful for initial screening, binding prediction algorithms cannot replace measurements of interacting molecules. Likewise, soluble refolding assays are exploited by many laboratories and prove very informative for molecular binding strength analyses, yet they might misrepresent the native biophysical interactions occurring at the cell surface between antigenic peptides and HLA molecules. To quantify the peptide:HLA binding strength more precisely in a cellular context, we developed a novel and simple competition assay termed “blue peptide assay”. The technology is based on the principles published by Kessler et al., who used 5-(iodoacetamido)fluorescein labelled reference peptides restricted to specific HLAs to assess the binding strength of selected, unlabeled competing peptide variants (33). For our assay, we synthetized cyanine 5-labeled HLA-A2-restricted Hepatitis B virus (HBV) cAg_18-27_ (FLPSDC*-(Cy5)-FPSV) decamers – the so-called blue peptide – as our positive, reference peptide (**Figure 1B**). HLA-A2-restriction and binding of the HBV cAg_18-27_ blue peptide was confirmed on T2/HLA-A2 ^pos^ and absent on C1R/HLA-A3 controls (**Figure 1C**). Using pairs of HLA-A2^pos^ and HLA-A2^neg^ cells tested in parallel, we determined both the binding kinetics (peptide:HLA association k_on_ = 2.3 x 10^4^ M^-1^ • s^-1^ and dissociation k_off_ = 1.2 x 10^-3^ s^-1^ rates) and the equilibrium dissociation constant (K_D_ = 52.6 nM) of the reference blue peptide, enabling precise quantification of strong and low HLA binders by competition binding (defined thereafter as peptide:HLA K_i_ affinity) (**Figure 1D**). To validate our technology, we first assessed the competition strength of native Melan-A/MART-1_26-35_-specific EAAGIGILTV peptide and its P2 ELAGIGILTV analog variant, reported to bind with superior affinity to HLA-A2 (54). As expected, both peptides displaced the reference blue peptide in a concentration-dependent manner (**Figure 1E**, top), with an exception seen at highest 50 μM concentration for ELA, possibly due to saturation-associated peptide-HLA instability. The modified ELA variant outcompeted the blue peptide at lower concentration than the native EAA peptide, reflecting a cell-based peptide:HLA K_i_ affinity that was 9 times stronger (variant ELA=0.52 μM and native EAA=4.67 μM). We then assessed all 17 selected HLA-A*02-restricted variants derived from the native Melan-A/MART-1_26-35_ peptide (**Figure 1E** bottom, Supplementary **Figure 1C** and data not shown), plus 21 variants originating from NY–ESO-1_157-165_ (Supplementary **Figure 1D** and data not shown). For Melan-A/MART-1_26-35_ variants, the inhibition equilibrium dissociation K_i_ constants ranged from 59 μM (low affinity n°5 variant EAAGIGILTM) to 0.37 μM (high affinity n°6 variant EMAGIGILTI), reflecting a 160-fold difference (**Table 1**). For NY-ESO-1_157-165_, the K_i_ constants were generally stronger, ranging from 2.4 μM (low affinity n°110 variant SALMWITQM) to 0.15 μM (high affinity n°116 variant SMLMWITQI) and reflecting a 16-fold difference (**Table 2**). Those K_i_ values correlated with the corresponding NetMHC4.0/4.1, NetMHCpan-4.0 indexes, the IEDB Consensus and PRIME2.0 percentile ranks, as well as with the *OD* values obtained during molecular refolding (**Figure 1F**, **Supplementary Figure 2B** and data not shown), indicating a good reliability of our assay. For both specificities, the range of the K_i_ values was larger than that obtained with the cell-free refolding assay, especially for the Melan-A/MART-1_26-35_ variants (1000x greater for Melan-A/MART-1 and 1.5x greater for NY-ESO-1), suggesting higher sensitivity and separation strength for the cell-based quantification method. Overall, we show that the “blue peptide” competition assay represents an accurate cellular method for characterizing surface-based peptide:HLA molecular affinity.

### Impact of peptide:HLA K_i_ affinity on T cell priming and function in patients vaccinated with native or analog Melan-A ^MART-1^ peptide

**Figure 2:**
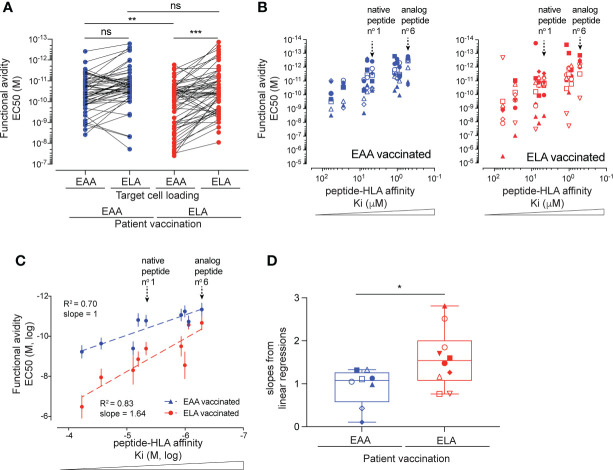
Functional avidity of tumor antigen-specific CD8 T cell clones derived from patients vaccinated with native EAA or analog ELA Melan-A peptides, and tested with K_i_ HLA-A2 affinity-enhanced variants. **(A)** Comparison of functional avidity (EC_50_) of Melan-A-specific T cell clones obtained from patients vaccinated with native EAA (n=31) or analog ELA (n=26) peptide and tested in criss-cross experiments against native (EAA) and analog (ELA)-pulsed targets. **(B)** Association analysis between functional EC_50_ avidity of T cells isolated from EAA (left, n=8) or ELA (n=10) vaccinated patients and K_i_ affinity values of the target peptide variants. Symbols represent individual T cells clones isolated from four (EAA vaccine), respective five (ELA vaccine) treated melanoma patients. **(C)** Correlation analysis for both EAA (blue) or ELA (red) vaccinated patients between the mean EC_50_ values obtained from T cell killing assays against a given target variant and the K_i_ affinity values of the respective peptide variants (n = 8 to 10). Average R square and slopes of the linear best fit regression line are given. Native peptide n°1 and analog peptide n°6 are indicated. **(D)** Comparison of the individual slopes (ΔY/ΔX) obtained from the linear best fit regression lines of the distinct EAA and ELA vaccine-derived T cell clones. Whisker boxes (5^th^ and 95^th^ percentile) with individual points, means and error bars are shown. ns p > 0.05 and *p ≤ 0.05, **p ≤ 0.01, ***p ≤ 0.001.

Peptide : HLA affinity is known to represent an important biophysical parameter that modulates immunogenicity and T cell function (9, 29). To assess the degree of correlation between the blue peptide-derived K_i_ affinity and the responsiveness of T cells recognizing those variants, we took advantage of a unique collection of human Melan-A/MART-1_26-35_-derived CD8 T cell clones isolated during phase I vaccination trials in cancer patients (13). Vaccination was performed with an emulsion of IFA containing CpG together with either the native Melan-A/MART-1_26-35_ (EAAGIGILTV) or the HLA-A2 affinity-improved Melan-A/MART-1_26-35_ (ELAGIGILTV) antigenic peptide. Previous studies on both T cell cohorts demonstrated that *ex vivo*-derived T cells isolated after vaccination with the native EAA formulation, although recruited at lower frequency, had overall improved TCR affinity, resulting in better functional recognition of the native tumor antigen compared to the ELA-derived T cells (31, 55, 56). This functional advantage disappeared when stimulation was performed with the higher affinity ELA peptide, increasing responsiveness of both EAA and ELA vaccine-derived T cells to comparably high levels (31). We validated and consolidated those results by testing *ex vivo* 57 clones isolated from 9 melanoma patients vaccinated with either EAA (n = 4) or ELA (n = 5) peptides. In average, EAA vaccine-derived T cell clones had superior functional avidity for recognition of the native peptide (**Figure 2A**). To further decipher the characteristics of those two sets of patient-derived Melan-A/MART-1_26-35_ specific T cell clones, we assessed their capacity to recognize peptide variants with distinct K_i_ affinities for HLA, which could hypothetically represent naturally occurring escape epitopes. 18 representative clones isolated from the 9 vaccinated melanoma patients were selected, expanded and tested in parallel *in vitro* killing assays against 9 peptide variants having low, intermediate or high K_i_ affinity for HLA-A2 (**Figure 2B** and **Table 1**). Overall, clones from native EAA-vaccinated patients outperformed clones isolated from ELA-vaccinated patients, reacting in average between 5x (for the high affinity ELA peptides n°6) and 550x (for the low affinity peptide n°5 EAAGIGILTM) stronger in terms of functional avidity (EC_50_). This enhanced functional superiority was particularly visible with peptides in the lower K_i_ affinity range and accompanied by a lower number of outliers (**Figure 2B**). Consequently, EAA-derived clones displayed flatter best-fit regression lines (**Figure 2C**) and weaker slopes (**Figure 2D**) than ELA-derived clones, because EAA-derived clones responded strongly to all peptide variants even in the lower K_i_ affinity range (**Supplementary Figures 3A, B**). Despite these differences, positive correlations between peptide:HLA affinity (K_i_) and functional T cell avidity (EC_50_) were found for both EAA and ELA-derived clones, at the global (**Figure 2C**) and individual (**Supplementary Figure 3**) levels. Altogether, these results show that T cell clones derived from patients vaccinated with the native EAA peptide recognized a broader spectrum of peptide antigen variants than ELA vaccine-derived T cell clones, maintaining higher functional avidity capacities toward peptides with lower HLA K_i_ affinities.

### Patient-derived, natural tumor-primed Melan-A-specific CD8 TILN cells show heterogeneous capacities to recognize peptide variants

**Figure 3:**
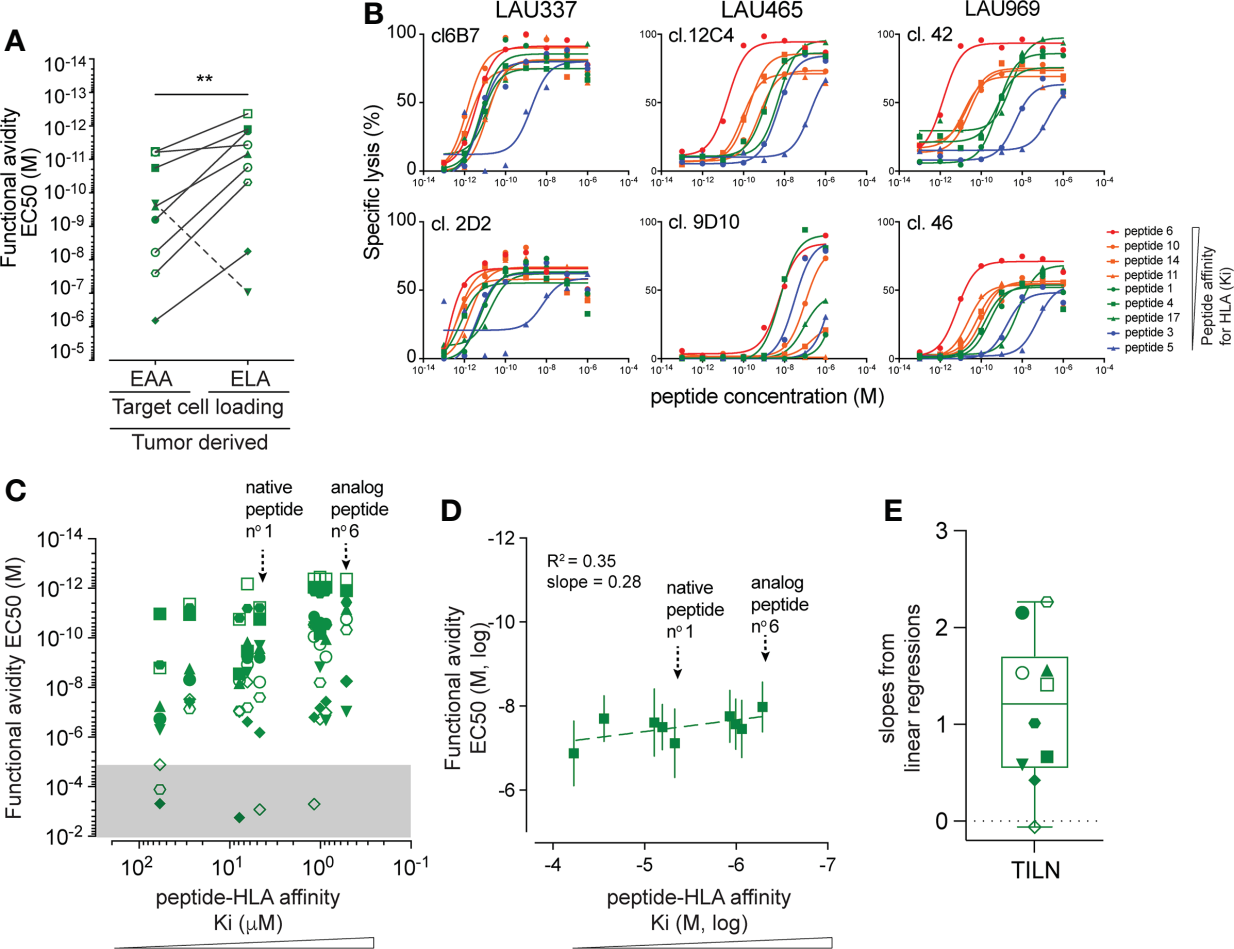
Characterization of peptide reactivity by naturally tumor-primed Melan-A-specific T cell clones. **(A)** Comparison of the functional avidity (EC_50_) of naturally tumor-primed Melan-A specific T cell clones (n=10) during parallel killing assays with targets presenting either native (EAA) and analog (ELA) peptide. Symbols represent individual T cells clones isolated from the TILNs of three untreated patients with natural Melan-A specific CD8 T cell responses. **(B)** Curves obtained from parallel cytotoxic killing assays of six clones isolated from three cancer patients against targets presenting nine Melan-A peptide variants with increasing K_i_ affinity for HLA-A2. **(C)** Association analysis between functional EC_50_ avidity of TILN-derived clones and K_i_ affinity values of the target peptide variants. The EC_50_ values in the gray area represent very low functional avidity and were excluded from further analysis. Symbols represent individual T cells clones. Native peptide n°1 and analog peptide n°6 are indicated. **(D)** Correlation analysis between the mean EC_50_ values obtained from TILN-derived T cell killing assays against a given target variant and the K_i_ affinity values of the respective peptide variants (n = 10). Average R square and slopes of the linear best fit regression line are given. Native peptide n°1 and analog peptide n°6 are indicated. **(E)** Projection of the slopes (ΔY/ΔX) obtained from linear regressions of the individual TILNs-derived T cells clones. Whisker box (5^th^ and 95^th^ percentile) with individual points, mean and error bars is shown. **p ≤ 0.01.

It is known that T cells can be naturally primed and activated by tumor antigens-presenting APC within nearby lymph nodes (3). To detect the spectrum of responsiveness of endogenous, tumor-primed CD8 T cells towards epitope variants with different K_i_ affinities for HLA, we generated 10 Melan-A/MART-1_26-35_-specific T cell clones derived from TILNs of 3 patients with spontaneous immunological responses toward their melanoma. These clones were first characterized functionally in killing assays against cell targets pulsed with the native EAA and the analog ELA peptides. All except one tumor-primed CD8 T cell clone showed higher functional avidity when challenged with the high K_i_ affinity ELA variant (**Figure 3A**). By testing functional avidity of all individual clones with the entire panel of Melan-A/MART-1_26-35_ peptide variants, we found variable and broad EC_50_ values, spanning up to 6-log differences for individual TILN clones (**Figures 3B, C**). Fine comparison of the lysis curves obtained for TILN clones showed that they could be separated in three groups. T cell clones from group 1 reacted weakly to the K_i_ peptide variants (e.g. 9D10 and 6C5 from LAU465), while clones from group 2 were strongly reactive to all variants (e.g. 6B7 and 2D2 from LAU377) (**Figure 3B**, **Supplementary Figure 3C**). The third group of clones recognized with incremental EC_50_ avidity cells pulsed with peptides of increasing K_i_ affinities (e.g. 12C4 and 12F6 from LAU465; 42 and 46 from LAU969), indicating stronger association (**Figure 3B**, **Supplementary Figure 3C**). In line with our findings for EAA/ELA vaccine-derived Melan-A-specific clones, pooled data showed a global positive correlation between peptide:HLA (K_i_) binding strength and T cell functional avidity (EC_50_) (**Figure 3D**). However, as natural tumor-primed CD8 TILN clones showed much broader and variable reactivities toward the Melan-A/MART-1_26-35_ peptide variants (**Figure 3C**), resulting in heterogenous linear regression slopes (**Figure 3E**), the R square correlative value as well as the average best-fit slope were weaker (**Figure 3D**). In summary, the naturally derived, tumor-primed TILN clones showed a broad heterogeneity of response patterns, ranging from overall very poor responders (group 1) to peptide K_i_ affinity-dependent (group 3) and independent (group 2) responders when tested against a panel of K_i_ affinity-matured peptide variants.

### No hierarchical, but cumulative impact of peptide:HLA and TCR-peptide:HLA affinity on T cell responses

**Figure 4:**
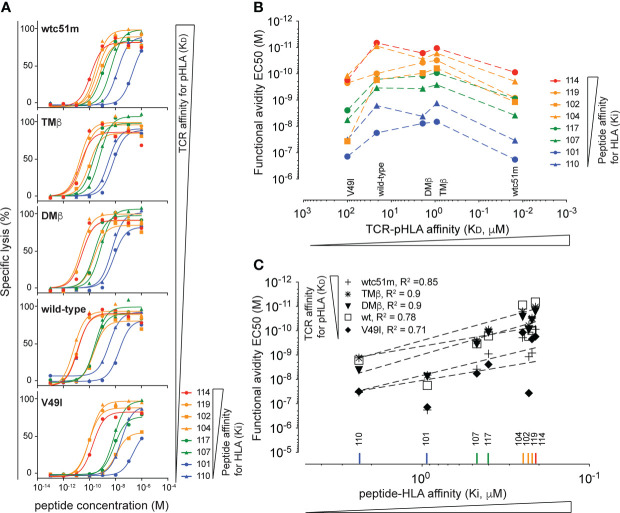
Hierarchical analysis of TCR-pHLA K_D_ affinity versus peptide:HLA K_i_ affinity. **(A)** Curves obtained from parallel cytotoxic killing assays of primary CD8 T cells expressing five NY-ESO-1_157-165_-specific TCRs of incremental affinities (very low affinity K_D_ of V49I < 100μM, wild type K_D_ = 21.4 μM, optimized DMβ = 1.91 μM and TMβ = 0.91 μM and very high affinity TCR wtc51 = 0.015 μM) against 8 NY-ESO-1_157-165_ peptide variants of increasing (n = 4 to 8). **(B)** Functional avidity values (EC_50_) obtained from the parallel killing assays of the NY-ESO-1_157-165_-specific T cells plotted against the K_D_ affinity of the TCR for the pHLA. **(C)** Correlation analysis between the functional avidity values (EC_50_) of the NY-ESO-1_157-165_-specific T cells and the K_i_ affinity of the respective target peptides. R square values are displayed.

The affinity of the TCR for the antigenic peptide:HLA complex (TCR-pHLA) also impacts on CD8 T cell function. To identify a possible hierarchical dominance between peptide:HLA K_i_ and TCR-peptide:HLA K_D_ affinities in the control of T cell functional avidity, we took advantage of a panel of primary CD8 T cells expressing NY-ESO-1_157-165_-specific TCRs isolated from LAU155, a long-term melanoma survivor (57) and engineered from the natural TCC9 Vb13.1 TCR to have incremental affinities for the peptide:HLA complex (44, 45). Recapitulating previous findings, we found that stimulation of those TCR-engineered T cells defined a functional bell shape curve along the TCR-pMHC affinity axis, with maximal functional avidity arising from the DMβ and TMβ TCR variants, known to be of optimal affinity for the NY-ESO-1:HLA antigen (**Figures 4A, B**, see wild-type peptide n° 101 EC_50_ curves). Five TCR-engineered CD8 T cells of incremental TCR affinity for peptide:HLA (K_D_) were stimulated with our panel of NY-ESO-1_157-165_ peptide variants of low, intermediate or high K_i_ affinity for HLA (**Figure 1A** and **Table 2**). We found that for each tested peptide variant (i.e. peptide 101 to 119), T cell functional avidity was systematically defined as a bell shape curve along the TCR-pHLA affinity range, although starting at distinct functional levels (**Figure 4B**). In other words, for any given peptide variant used on target cells, T cells expressing very low and very high affinity TCRs consistently displayed a lower functional avidity than T cells expressing optimal-affinity TCRs (**Figure 4B**). Yet, when using peptide variants of higher HLA K_i_ affinity (i.e. variants 119 and 114), significant functional increases were detected for all TCR variants. In contrast to the impact of TCR-pHLA affinity (K_D_), the impact of the peptide:HLA affinity (K_i_) on T cell functional avidity was linear and generated positive correlations for all TCR-engineered variants (**Figure 4C**), in line with the results obtained in the Melan-A model (**Figures 2**, **3**). The functional impact of K_i_ affinity was quantitatively more important than the impact of TCR-pHLA K_D_ affinity. Indeed, peptide K_i_ differences of <10-fold (e.g. between peptide 101 and 114) were capable of augmenting the resulting T cell functional avidity up to 1000-fold, while variations in TCR K_D_ affinity of up to 1000-fold led to a maximal increase of 25-fold in functional avidity. Altogether, we confirmed in a second model that peptide:HLA K_i_ affinity quantitatively affects T cell function, with strong positive correlations, contrasting to the qualitative impact of TCR-pHLA K_D_ affinity. By performing such criss-cross experiments, we now uncovered that there is no hierarchical dominance of one affinity parameter (peptide K_i_ or TCR K_D_) over the other, but rather cumulative - positive and negative - effects within the trimolecular TCR-peptide:HLA complex, all contributing to the overall T cell function.

## Discussion

Responses of CD8 T cells initially depend on intricate biophysical interactions occurring within the TCR-peptide:HLA trimolecular complex at the interphase of T cells and antigen presenting cells. Both the affinity - or binding strength - of the antigenic peptide within the groove of the HLA, and that of the TCR to the peptide:HLA complex are key parameters that determine TCR activation, TCR signaling and T cell responsiveness. Here, we used the blue peptide competition assay and a step-by-step analysis of two tumor-reactive model systems in which both the peptide:HLA (K_i_) and the TCR-pHLA (K_D_) affinities can be selectively modified to interrogate their respective functional and hierarchical impact. We found a cumulative effect of both parameters on T cell responses, but no hierarchical dominance.

In the past, multiples technologies have been established with the specific goal to measure binding affinity of peptide to HLA class I and II molecules in the cellular context (58–62). Binding or killing competition assays helped ranking the relative binding strengths or the functional avidities of large series of antigen peptide variants, but none of these assays could provide accurate affinity values *per se* (33, 54). Consequently, we developed the “blue peptide” assay - a precise peptide:HLA competition assay - that enables flow cytometry-based and direct quantification of peptide:HLA affinity at the surface of T2 cells, combined to subsequent functional assays. Although focusing on HLA-A2-restricted ligands, the blue peptide assay can easily be translated to other HLA molecules by selecting corresponding fluorescent-tagged antigens restricted by HLA I or II complexes. Cyanine conjugation can be performed *a priori* on any solvent-exposed amino-acid side chain of an HLA bound peptide. Preferable are easily labeled residues (e.g. lysine and cysteine) outside the P1-3 and omega anchor positions (63). Choosing antigens with intermediate HLA affinity is optimal, since it enables precise calculation of equilibrium inhibition constant (K_i_ affinity) for virtually any unlabeled, native or engineered, weak or strong competitor ligand (64). The affinity value of the Hepatitis B virus (HBV) cAg_18-27_ [FLPSDC*-(Cy5)-FPSV] blue peptide to the HLA-A2 cleft has such characteristics (K_D_ =52.6 nM), as it falls at the exact threshold (50 nM) of peptides reported as weak or strong HLA binders (65).

We chose Melan-A/MART-1_26-35_ and NY–ESO-1_157-165_ antigens to validate our assay, because these are two important tumor antigens exploited in various active and passive tumor immunotherapy strategies. 17 Melan-A/MART-1_26-35_ and 21 NY–ESO-1_157-165_ peptide variants were synthesized with amino-acid substitutions at primary HLA anchor sites known to profoundly impact pHLA binding and consequent T cell function (14, 66, 67), resulting in HLA binding K_i_ affinities spanning their entire physiological range. Of course, it would have been interesting to substitute additional amino-acid within the 9/10mers since binding of peptides to HLA is typically a composite of interactions of (i) canonical N and C-termini anchors with the HLA protein, (ii) peptide side chains with allele-specific HLA binding pockets and (iii) secondary peptide side chains with HLA residues (68). With our collection of peptide variants, we obtained good correlations without any outliers between blue peptide-derived cellular K_i_ affinity and soluble molecular HLA refolding results, which remain the standard method for assessing molecular peptide:HLA binding strengths in solution. The blue peptide assay also proved very sensitive, especially for Melan-A variants, with K_i_ affinities ranging 3-log compared to 1 for the refolding test. As expected from the literature, we found positive correlations between peptide:HLA K_i_ affinity and functional avidity EC_50_ (i.e. T cell sensitivity to recognize and destroy tumors cell lines), both for patient-derived NY-ESO-1 and Melan-A/MART-1-specific clones. However, the fact that NY-ESO-1 peptide variant n°101 performed weaker than n°110, despite having a higher K_i_ affinity, a better refolding value and a better predicted avidity score, suggests that additional parameters besides the peptide:HLA interaction strength can influence T cell functional avidity. As such, other determinants including peptide structure and flexibility within the HLA groove and/or influence off the peptide:HLA complex on other surface immunomodulatory receptors might also tune T cell responsiveness (69).

Many bioinformatic tools and algorithms based on structural knowledge have been generated for the prediction of peptide:HLA binding, T cell immunogenicity and function (70, 71). Noteworthy, we found a correlation between the blue peptide-derived K_i_ affinity values and *in silico* algorithm indexes, yet with multiple outliers. Surprisingly, the native immunogenic Melan-A/MART-1_26-35_ EAA epitope would have been excluded by NetMHC4.0 algorithms based on its poor immunogenicity index. Likewise, an endogenous mouse neoantigen of very low affinity for MHC H2-K^d^ was also categorized as non-MHC I-binder and non-immunogenic, yet it was able to elicit CD8 T cell-dependent tumor rejection and protection (37). Discrepancies between *in silico* predictions and immunogenicity were also reported for multiple HLA-A2-resticted NY-ESO-1 peptides (39), prompting ongoing improvement of prediction tools (41). Our blue peptide assay, or other robust affinity measurement technologies should be used to validate bioinformatic predictions to screen for potentially immunogenic peptides.

To assess the impact of K_i_ affinity on immunogenicity of peptides in human, we took advantage of clinical trials in which cohorts of melanoma patients were vaccinated with formulations containing two different Melan-A/MART-1-specific peptides: the native low K_i_ affinity EAAGIGILTV peptide and the high K_i_ affinity ELAGIGILTV variant (13). Clearly, tumor antigen-specific CD8 T cells isolated from both cohorts showed increased functional avidity when stimulated with peptide variants of higher HLA-A2 K_i_ affinities. As reported previously (13), this study confirms that T cell clones derived from patients after vaccination with the native, low affinity EAA peptide were globally of higher avidity when compared to those obtained after vaccination with the high affinity ELA peptide. We also report that the EAA-specific CD8 T cells outperformed the ELA-specific ones for recognition of a variety of single or double amino-acid substituted peptide variants, especially those having lower HLA K_i_ affinities. This higher cross-recognition potential against peptides with subtle amino-acid and HLA-binding changes might arise from the overall broader TCR β-chain repertoires and lower CD8-dependency found in T cells after EAA versus ELA vaccination (31, 72). Structural analysis and molecular dynamic simulations in the HLA-A2 and other systems revealed that peptide binding to HLA I molecules can induce significant and diverse allosteric changes, creating molecular plasticity that can critically influence antigen recognition by T cells and impact cross-reactivity (73, 74). Specifically, the presence of the weak alanine anchor residue in the native EAA peptide was shown to afford a larger number of pHLA conformers than the analog ELA, including configurations that enable induced fit, high affinity interactions with the TCR (75, 76). Together, our findings reinforce the idea that T cells raised against modified, high K_i_ affinity variants (e.g. ELA) might be more specific to the corresponding non-native mutated peptide, and are unable to efficiently recognize tumors expressing the native or lower affinity peptides (66).

Collectively, compared to the analog vaccination settings, tumor-reactive CD8 T cells recruited after vaccination with the native EAA peptide are of reduced frequency (13), yet they show a higher T cell clonotype diversity with subtle CDR3β structural differences (72), have TCRs of higher structural avidities linked to CD8-independancy and enhanced function (31), and are in average more cross-responsive to epitope-focused peptide variants (1 or 2 amino-acid substitutions from the wild-type sequence) having widespread K_i_ affinity (this study). These beneficial T cell characteristics must initially arise by favorable intrinsic peptide:HLA and TCR-pHLA-associated biophysical mechanisms impacting T cell clonotypic recruitment and functional potential. We propose that weaker HLA binding of the native EAA peptide results in the requirement of stronger TCR binding to pHLA to reach sufficiently strong immunogenicity for T cell activation. Thus, vaccination with the native EAA peptide is likely to select higher avidity T cells during priming and/or boosting. For vaccine formulations, our findings pinpoint to the use of low to intermediate affinity peptides that minimally derive from the structure of the corresponding native targets to maintain reactivity of the recruited T cells to the native tumor epitope. If the native peptide is not immunogenic, new strategies with careful structural evaluation of peptide:MHC interactions should be developed to generate peptide variants with increased MHC affinity that generate T cells with strong cross-reactivity toward the native, unmodified low affinity tumor-associated peptide (77).

We succeeded in isolating dozens of natural tumor-primed, Melan-A/MART-1-specific T cell clones derived directly from TILNs of cancer patients. By testing them against the native Melan-A/MART-1 EAA peptide and the eight Melan-A peptides variants with distinct K_i_ affinities for HLA-A2, we found that TILN cells were versatile both in their recognition capacities and cross-reactivity profiles toward the other variants. We found up to 10’000-fold variation in wild-type EAA-pulsed target recognition capacities between TILN clones. As detected in the vaccination settings, most clones performed better when triggered by peptides with higher K_i_ affinities, with some exceptions. LAU465 clone 9D10, for instance, performed best against the native EAA and the ELA peptide, but failed in global recognition of other Ki affinity variants (group 1). Some clones (e.g. LAU969 clone 42) generated steep regression slopes (group 3), indicating strong specific biases and preferential reactivity toward peptides with high HLA-A2 K_i_ affinities, reminiscent of the profiles detected with ELA vaccination-induced T cells. Other clones (group 2) generated elevated and flat regression lines (e. g. LAU969 clone 14) like T cells obtained after vaccination with low affinity EAA peptide, suggesting higher cross-reactive potential toward peptide variants. As such, additional studies based on clonotype repertoires and CDR3 structural patterns relative to the T cells induced by the two types of vaccines (72) and whether their TCR-pHLA affinity correlates with functional avidity and/or off-target cross-reactivity are highly relevant, but are beyond the scope of this manuscript. Moreover, we used standard SPR and reversible multimers technology to assess TCR-pHLA structural affinities (31, 78), however, it would also be interesting to see how other binding parameters, including 2D affinity (79, 80) and catch-bond formation (81), would categorize those vaccine- versus tumor-derived CD8 T cells. We believe that the epitope-focused cross-reactivity detected in tumor-derived T cells might be favorable in checkpoint blockade therapy, where T cell clonotypes with such diverse functional profiles would become reactivated and recognize target cells expressing native or escape epitopes, providing clinical benefit.

An important finding of our study is that there is no hierarchical dominance of peptide:HLA K_i_ affinity or TCR-pHLA K_D_ affinity in terms of functional avidity, but rather cumulative inputs, contributing to overall T cell response. By testing the NY-ESO-1 peptide variants on our panel of TCR-engineered, NY-ESO-1-specific T cells, we show that both biophysical parameters provide cumulative inputs to overall T cell function. Quantitatively, T cell functional avidity is largely and linearly dependent on peptide K_i_ affinity for HLA, yet it is qualitatively calibrated by the TCR K_D_ affinity for pHLA complexes, which seems to control the extend and sensitivity range of T cell responses. Furthermore, our findings indicate that the functional hyporesponsiveness linked to nonspecific chronic interactions between HLA and very high affinity TCR (e.g. wtc51m) (20) can be readily reversed by peptides with high K_i_ affinity for HLA. In other words, increased peptide:HLA K_i_ affinity on the target side of the trimolecular TCR-pHLA complex can overcome preexisting TCR affinity-associated T cell functional impairments. The rapidity of this reversal (4h for a cytotoxic assay) argues against epigenetic or transcriptional/translational control of such T cell hyporesponsiveness. Our current findings are in line with previous observations showing that very high affinity TCR signaling and associated function can be partially restored with high concentration of native target peptide (44, 82). Collectively, these data indicate that this type of functional impairment is likely controlled very proximally, at the TCR complex itself through post-translational modifications, potentially by phosphorylation and/or ubiquitination events on proximal signaling molecules (83).

Our study suggests that vaccination with native, intermediate K_i_ affinity peptides, which induce tumor antigen-specific CD8 T cell clonotypes of high functional avidity and killing capacity should be favored because they generate better recognition of putative tumor-derived, neo-antigen-like targets, which might become escape variants. Yet, careful analysis of the responding TCRs should be performed to avoid toxic cross-reactivity against tumor-irrelevant epitopes. In that regard, Karapetyan et al. studied the clinically-relevant high-affinity NY-ESO-1^c259^ TCR, and performed functional assessment of epitope variants bearing all possible substitutions at each position, associated to algorithm-based prediction for genome-wide off-target activity, to select safe therapeutic epitopes and TCRs (84). Alternatively, careful engineering of tumor-specific TCRs with modified bond lifetime to pMHC enabled selection of highly potent TCRs, while maintaining overall low affinity and avoiding adverse cross-reactive events (24). Dissecting the contribution of both TCR-pHLA and peptide:HLA parameters within the overall TCR-peptide:HLA hetero-trimeric complex and understanding the biophysical and biological rules generating safe, efficient and protective T cell responses has clear implication in the field of cancer immunotherapy. Our stepwise analysis of those biophysical variables will help to identify and generate optimal therapeutic peptides and TCRs.

## Data availability statement

The original contributions presented in the study are included in the article/**Supplementary Material**. Further inquiries can be directed to the corresponding authors.

## Ethics statement

This study was reviewed and approved by the Ethics Committee for Clinical Research of the University of Lausanne. The patients/participants provided their written informed consent to participate in this study.

## Author contributions

Study design: PB, JS, DS, NR and MH. Acquisition of data: PB, JS, CC, NB and MH. Analysis and interpretation of data: PB, JS, CC, PG, IL, DS, NR and MH. Writing, review and/or revision of the manuscript: PB, JS, DS, NR and MH. All authors contributed to the article and approved the submitted version.

## Funding

This study was sponsored and supported by the Department of Oncology (University of Lausanne), the ISREC Foundation (Switzerland), and the Swiss National Science Foundation (310030-179280). Open access funding was provided by the University of Lausanne.

## Acknowledgments

We are grateful to Anne Wilson, Romain Bedel and Danny Labes for operational support at the flow cytometry facility of Lausanne, to the tetramer and peptide core facility of the UNIL/CHUV for peptide and multimer synthesis and for quality controls. We are also thankful to Nicole Montandon and Natasa Jovanovic for their proficient technical and secretarial help.

## Conflict of interest

The authors declare that the research was conducted in the absence of any commercial or financial relationships that could be construed as a potential conflict of interest.

## Publisher’s note

All claims expressed in this article are solely those of the authors and do not necessarily represent those of their affiliated organizations, or those of the publisher, the editors and the reviewers. Any product that may be evaluated in this article, or claim that may be made by its manufacturer, is not guaranteed or endorsed by the publisher.

## Supplementary material

Supplementary Figure 1 Functional substitution scan and competition assay. **(A)** Representative example of a cytotoxic killing assay obtained with a Melan-A_26-35_-specific T cell clone and targets pulsed with Melan-A native or variant peptides bearing amino-acid changes at anchor position 2/9 (**B)** Alanine scan cytotoxic killing assay obtained with a NY-ESO-1_157–165_-specific T cell clone and targets pulsed with a titration of NY-ESO-1 native (in blue) or alanine-substituted peptide variants (P2 and P9 substitution curves are shown in green and orange, respectively). (**C)** Representative example of the cell-based blue peptide competition assay showing titration curves obtained for the different K_i_ affinity peptide variants. The gMFI decrease along the competitor peptide concentration reflects the competition of the peptide variant with the “blue”-HBVc reference peptide, allowing to determine the half-maximal inhibitory concentration (IC_50_) and the competition-derived affinity K_i_. (**D)** Representative example of the cell-based blue peptide competition assay performed with distinct NY-ESO-1 peptide variants.

Supplementary Figure 2 Correlation analysis between PRIME2.0 and peptide:HLA affinity **(A)** Correlation between PRIME2.0 and binding assay refolding OD indexes. Melan-A_26-35_ (left graph) and NY-ESO-1_157-165_ (right graph) peptide variants are shown with the respective native and analog peptides highlighted in blue and red. R square and p values were obtained from Pearson correlation analysis. **(B)** Correlation between K_i_ affinities (μM) obtained from the cellular blue peptide competition assay and percentage rank values obtained from the PRIME2.0 algorithm for both NY-ESO-1 (left) and Melan-A/MART1 (right) variants. Native and analog peptides are shown in blue and red for both peptides, respectively.

Supplementary Figure 3 Correlation analysis between functional avidity and peptide:HLA affinity.**(A, B**) Correlation analysis between functional avidity EC_50_ values and peptide:HLA K_i_ affinity from the individual cancer patient-derived T cell clones isolated after EAA or ELA vaccination. (**C**) Correlation analysis between functional avidity EC_50_ values and peptide:HLA K_i_ affinity from the individual cancer patient-derived T cell clones isolated from TILNs. Individual R square values and slopes from best fit linear regression lines are indicated.
