# Erratum: CD8 T cell function and cross-reactivity explored by stepwise increased peptide-HLA versus TCR affinity

**DOI:** 10.3389/fimmu.2023.1178295

**Published:** 2023-03-14

**Authors:** Frontiers Production Office

**Affiliations:** Frontiers Media SA, Lausanne, Switzerland

**Keywords:** cancer immunotherapy, vaccine peptide, melan-A/MART-1, NY-ESO-1, peptide-HLA binding affinity, TCR-peptide-MHC affinity, human CD8 T cells

An omission to the funding section of the original article was made in error. The following sentence has been added: “Open access funding was provided by the University of Lausanne”.

The original version of this article has been updated.

