# Supplementary figures and images for: CD8 T cell function and cross-reactivity explored by stepwise increased peptide-HLA versus TCR affinity
Source: Front Immunol. 2022 Aug 10;13:973986. doi: 10.3389/fimmu.2022.973986 (PMC9399405; doi:10.3389/fimmu.2022.973986)

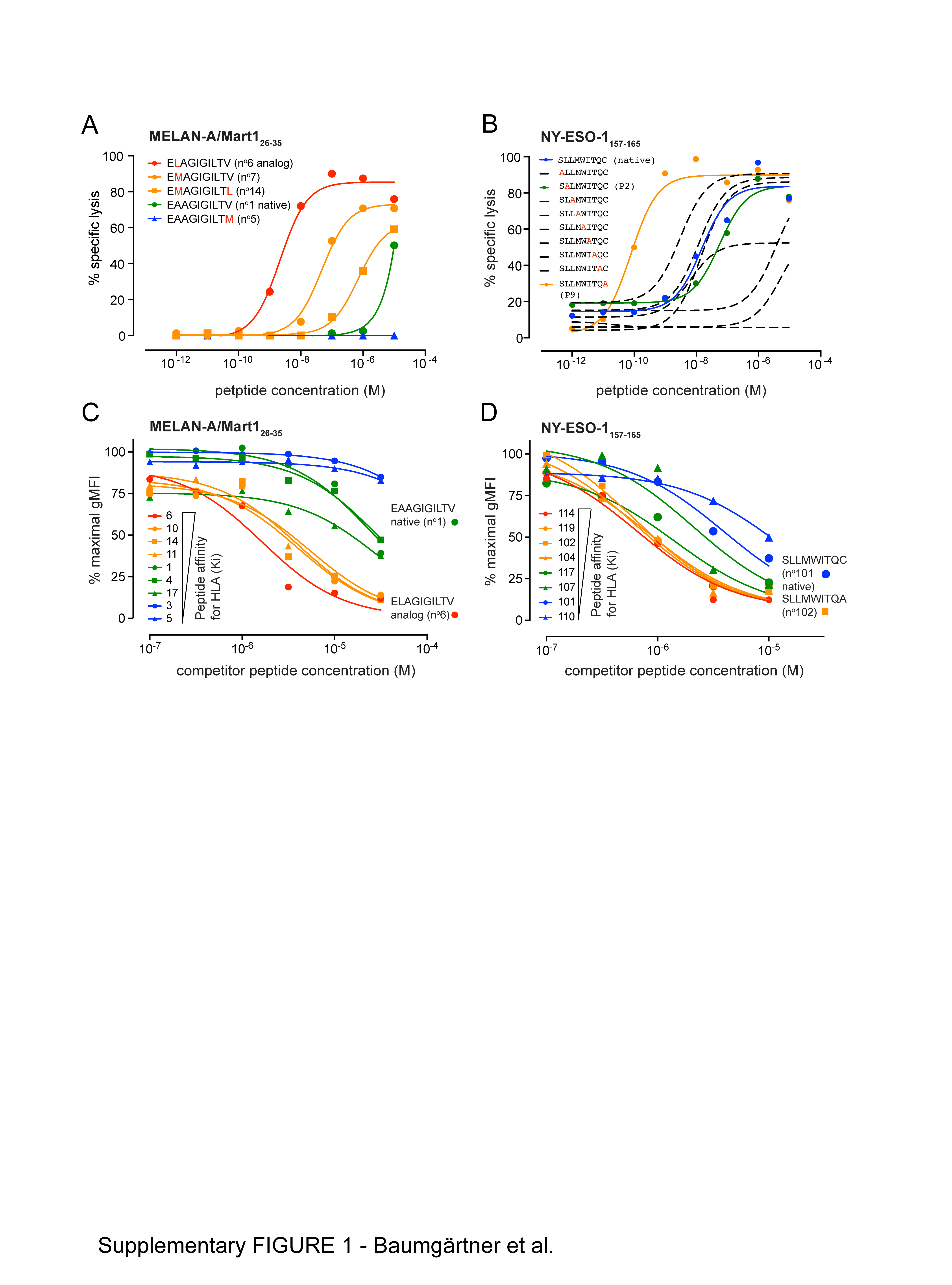

Supplement: Supplementary Figure 1 — Functional substitution scan and competition assay. (A) Representative example of a cytotoxic killing assay obtained with a Melan-A26-35-specific T cell clone and targets pulsed with Melan-A native or variant peptides bearing amino-acid changes at anchor position 2/9 (B) Alanine scan cytotoxic killing assay obtained with a NY-ESO-1157–165-specific T cell clone and targets pulsed with a titration of NY-ESO-1 native (in blue) or alanine-substituted peptide variants (P2 and P9 substitution curves are shown in green and orange, respectively). (C) Representative example of the cell-based blue peptide competition assay showing titration curves obtained for the different Ki affinity peptide variants. The gMFI decrease along the competitor peptide concentration reflects the competition of the peptide variant with the “blue”-HBVc reference peptide, allowing to determine the half-maximal inhibitory concentration (IC50) and the competition-derived affinity Ki. (D) Representative example of the cell-based blue peptide competition assay performed with distinct NY-ESO-1 peptide variants. [file Image_1.tif]

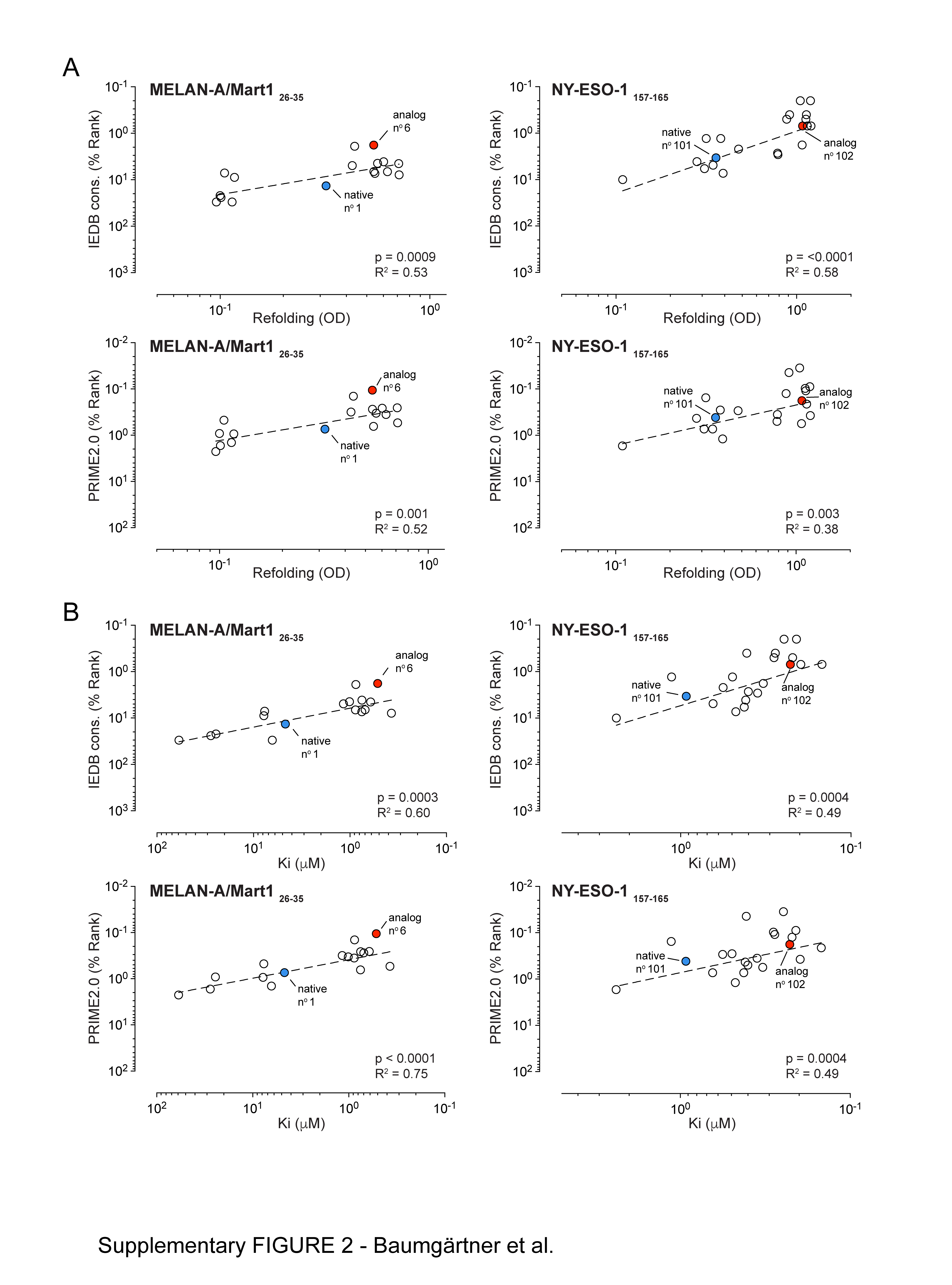

Supplement: Supplementary Figure 2 — Correlation analysis between PRIME2.0 and peptide:HLA affinity (A) Correlation between PRIME2.0 and binding assay refolding OD indexes. Melan-A26-35 (left graph) and NY-ESO-1157-165 (right graph) peptide variants are shown with the respective native and analog peptides highlighted in blue and red. R square and p values were obtained from Pearson correlation analysis. (B) Correlation between Ki affinities (μM) obtained from the cellular blue peptide competition assay and percentage rank values obtained from the PRIME2.0 algorithm for both NY-ESO-1 (left) and Melan-A/MART1 (right) variants. Native and analog peptides are shown in blue and red for both peptides, respectively. [file Image_2.tif]

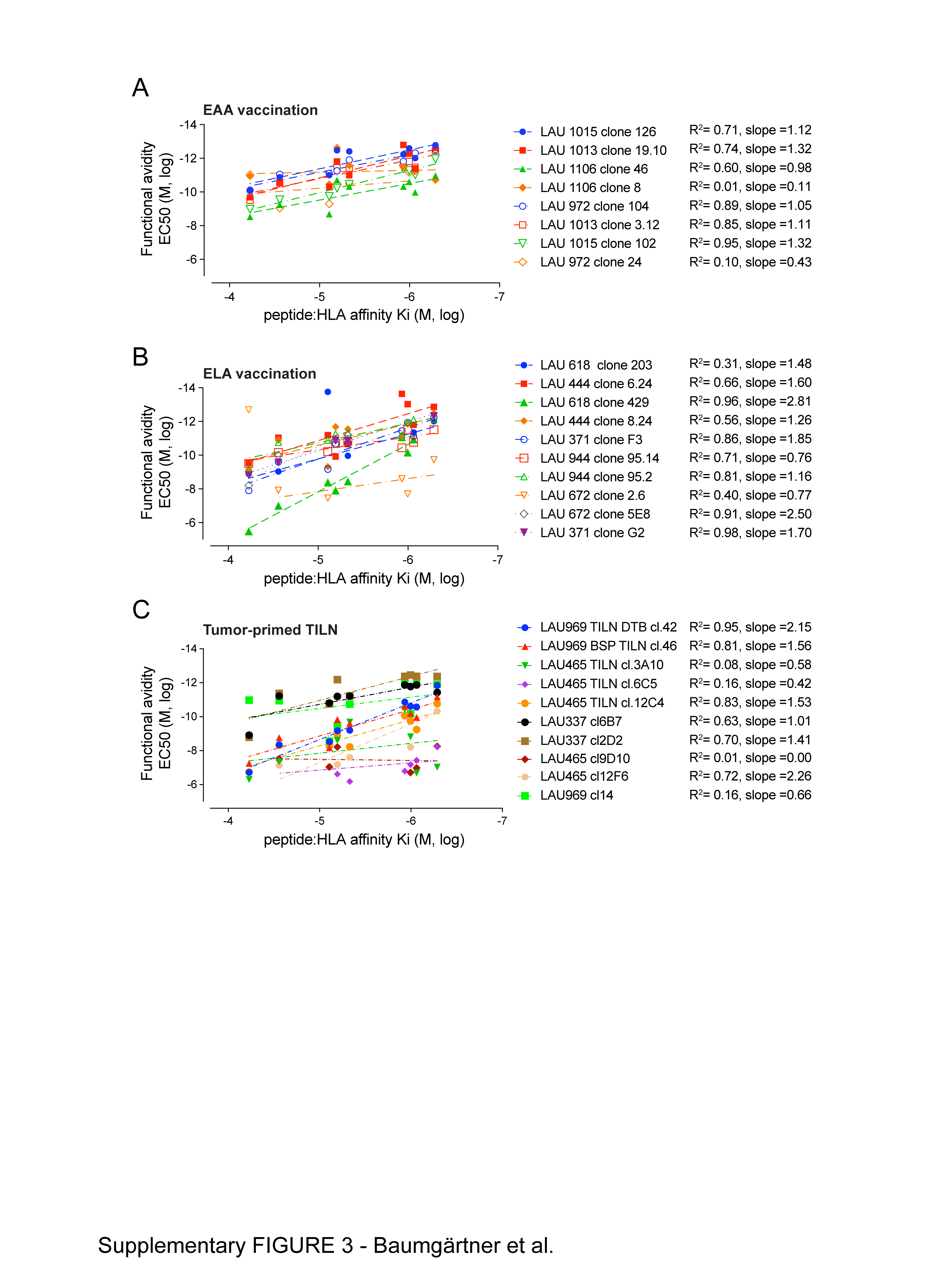

Supplement: Supplementary Figure 3 — Correlation analysis between functional avidity and peptide:HLA affinity.(A, B) Correlation analysis between functional avidity EC50 values and peptide:HLA Ki affinity from the individual cancer patient-derived T cell clones isolated after EAA or ELA vaccination. (C) Correlation analysis between functional avidity EC50 values and peptide:HLA Ki affinity from the individual cancer patient-derived T cell clones isolated from TILNs. Individual R square values and slopes from best fit linear regression lines are indicated. [file Image_3.tif]
